# Women’s experience of continuity of midwifery care in North-Eastern Italy: A qualitative study

**DOI:** 10.18332/ejm/159358

**Published:** 2023-02-15

**Authors:** Stefania Poggianella, Elisa Ambrosi, Luigina Mortari

**Affiliations:** 1 Independent Midwife, Trento, Italy; 2 Department of Diagnostics and Public Health, University of Verona, Verona, Italy; 3 Caring Education Research Center, Department of Human Sciences and Department of Diagnostics and Public Health, University of Verona, Verona, Italy; 4 Center of Educational and Didactic Research, Department of Human Sciences, University of Verona, Verona, Italy

**Keywords:** continuity of care, midwifery-led care, personalization of care, COVID-19, qualitative research, midwifery assistance

## Abstract

**INTRODUCTION:**

The establishment of a maternity path is often hampered by the fragmentation of care processes resulting in discontinuity of care. The interruption of continuity of care negatively affects the experience of maternity. The purpose of this research is to analyze the experience of women who get midwifery continuity of care from pregnancy till after childbirth.

**METHODS:**

A qualitative study using a phenomenological-grounded approach was undertaken. Audio-recorded semi-structured interviews were taken from 11 pregnant women who received midwifery care during maternity. This research was carried out between March 2020 and February 2021.

**RESULTS:**

Continuous and quality care is what allows women to develop new skills, increasing awareness and confidence in themselves and in their abilities both during pregnancy and after delivery. Assistance provided by competent professionals allows women to be taken in charge globally with greater personalization of care.

Since the research was carried out during the first wave of the COVID-19 pandemic, some of the repercussions that the situation had on women during maternity were also experienced negatively, such as the interruption of continuity of care or the inability to choose whom to have next to.

**CONCLUSIONS:**

From the perspective of prevention and protection of maternal and child health, in the short- and long-term, it becomes essential to focus on developing maternal competencies. This may be possible by implementing midwifery continuity of care pathways with an appropriate and flexible organizational system capable of responding to women’s needs throughout the maternity journey, even during periods of a health emergency.

## INTRODUCTION

Midwifery continuity of care is expressed at the level at which a care pathway is experienced as integrated and consistent with the patient’s medical needs and personal context^1^. Two fundamental elements must coexist to speak of continuity of care: care addressed to a person that lasts over time^1^.

Haggerty et al.^1^ summarize the concept of continuity in three levels: continuity of information (informational continuity), management continuity, and relational continuity.

In the obstetric-neonatal area, continuity of care provided throughout the maternity path by the same professional figure is an organizational form that makes it possible to overcome the segmentation of interventions and to offer women unified care^2^. It responds to women’s primary need, since motherhood places them in a context of continuous physical and psychological change.

The World Health Organization recommends that birth pathway care ensures a healthy mother and baby with the lowest possible level of care compatible with safety^3^.

The midwifery-led model of care is reliable and safe, and the literature shows the importance of considering this model of care within health services^4,5^. The therapeutic continuity provided by the midwife acts by placing the woman/couple at the center, offering containment, and making the woman feel competent^5^. For women at low risk of complications, caseload midwifery has been shown to increase women’s satisfaction with antenatal, intrapartum, and postpartum care^6^. Women were more likely to report that midwives kept them informed, they were given an active say about decisions, their worries were taken seriously, and care was provided safely and competently. Moreover, midwives were encouraging, reassuring and emotionally supportive^6^. The caseload midwifery model was associated with more spontaneous vaginal births, less intrapartum analgesia, and fewer episiotomies^7^. This model also effectively reduces the cesarean section rate in settings with a relatively high baseline cesarean rate^7^.

In Italy, organizational model change is currently underway to provide care models for women with low-risk pregnancies and labor/childbirth. By now, the predominant care model is that of the private gynecologist, chosen by 66% of women^8^. The ‘Guidelines for the definition and organization of autonomous care by midwives for low-risk pregnancies’^9^, issued by the Ministry of Health in 2017, provide for establishing and activating functional areas for low-risk childbirths in all birth points. The aim is to ‘promote organizational solutions that not only meet quality and safety criteria but also guarantee greater continuity of care in pregnancy, childbirth and puerperium, offering the woman, duly informed, the possibility of choosing the care setting’^9^.

Generally, these organizational models still need to be improved in the country. The birth path suffers from a fragmentation of the care process, creating a discontinuity of care. The woman finds herself interfacing with different professionals during pregnancy, birth, and puerperium^5^. However, these organizational models are present in some regions.

Nevertheless, despite the pathways present, continuity of care is only sometimes guaranteed. Often women are followed in continuity during pregnancy but not during labor/childbirth or after delivery. In other cases, they are followed during pregnancy and after childbirth. In contrast, in just a few cases, women are followed by the same midwife during pregnancy, labor/delivery and after childbirth.

The situation has further changed due to the COVID-19 pandemic. The coronavirus pandemic officially hit Italy on 30 January 2020, and, despite containment measures, a complete lockdown state was declared between 8 March and 3 May 2020^10^. On 11 March, the World Health Organization declared a global pandemic status^11,12^ and maternity services underwent rapid changes^13^. Hospitals activated telemedicine services and instituted restrictive visiting policies that did not allow support persons, including the woman’s partner, to be physically present in maternity wards during pregnancy, labor and postpartum, allowing access only during delivery^14^.

This research aims to analyze in depth the experience of women who get midwifery continuity of care, investigate their emotional state, thoughts and needs, and attempt to understand their most intimate and profound meanings from pregnancy until after the baby’s birth. Attention is also paid to the differences that might emerge between different care models (women followed in continuity only during pregnancy, women followed in continuity during pregnancy and after childbirth but not during delivery, and, finally, women followed in continuity during pregnancy, delivery and after childbirth).

## METHODS

### Design and setting

This qualitative research was carried out across a region in North-Eastern Italy where, among other maternity services, a path has been activated that allows pregnant women at low risk of complications to receive the assistance of a midwife who acts as the point of reference throughout the entire period of pregnancy and in the first weeks after childbirth. This is a service provided by both the public and private health systems. In case of need, midwives collaborate with a team of professionals, including doctors, psychologists and social workers.

The continuity model provided by the public system is a 9 a.m. to 6 p.m. model, Monday to Friday. At the same time, the model provided by private service is one with continuous midwifery availability also at nights, weekends, and public holidays.

### Sampling

Women were recruited who were guaranteed continuity of care by the same midwife (whether working in the public or private sector) for at least the duration of their pregnancy. The continuity could extend until labor/delivery and after birth. Sampling was performed purposefully for the midwives who could propose to their clients to participate in the research and through the snowball technique. The sample was obtained by applying the following inclusion criteria: aged >18 years, before 24 weeks of pregnancy (time of the first interview), and understanding of the Italian language. All pregnant women who met the inclusion criteria were invited to participate. The purpose and modalities of the research were explained to all the women and were assured that their ongoing care would not be affected by participation. The women were also assured that what resulted from the interviews would not be shared and that the data would be anonymized. Participants were also informed about the possibility of leaving the study at any time without compromising the quality of care.

Women willing to participate agreed to leave a phone number through which they were contacted for the interview by the researcher. This research was carried out between March 2020 and February 2021 and involved 11 women. Eighteen women were eligible to participate, but seven women declined participation due to the commitment required by the study. Of the 11 women interviewed, ten replied to all three planned interviews. In contrast, one woman replied only to the first interview, choosing, during the process, to no longer be followed by the midwife of reference. A total of 31 interviews were collected.

### Data collection

This research was carried out using semi-structured interviews. Such modality allows for free expression of thoughts, states of mind and concepts considered necessary by the interviewee, and investigates them in depth.

The same researcher conducted all interviews after receiving the approval of a methodological expert who assessed an initial pilot interview.

Three interviews were carried out with each woman, one at the end of the second trimester (24–26 weeks of pregnancy), one at the end of the third trimester (36–38 weeks of pregnancy) and one after childbirth (between the 10th and 30th day after birth). The interviews were designed to assess the women’s experience during the different stages of care and their relational dynamics with the midwife.

The questions were carried out following an outline initially prepared to anchor the questions as much as possible to significant episodes (Table 1).

**Table 1:** Questions used as guidelines during interviews


How does/did you feel about being accompanied during pregnancy?
Do you remember any recent occurrence or any recent concern regarding pregnancy that prompted you to contact your midwife?
Do you remember how you felt? What thoughts did you have at that moment?
How did your feelings change? How did your thoughts change?
During this maternity journey is there anything particularly significant that you would like to highlight and/or anything that you missed, that you felt you needed?
If you had to represent this accompaniment along the pregnancy by using an image, a word or a thought, what would that image, word or thought be?

Each interview lasted on average about 25 minutes and was conducted in Italian. To ensure that the English translation reflected the true meaning of the women’s words, the quotes were first translated from Italian to English and then back-translated from English to Italian by a different official translator.

Due to the health emergency from COVID-19, the interviews were performed via telephone, audio-visual or audio-only connection.

After the researcher received the participants’ written consent, the interviews were recorded using an audio device. Before consent was given, the purpose of the recording was explained to the women. It was also ensured that only the researcher could access the recording, and all data would be anonymized. The women were informed that they could withdraw their consent by simply informing the researcher.

Women were invited to participate until data saturation occurred at the last interview.

The interviews were analyzed using the phenomenological-grounded method, according to Mortari^15^. This method involves a repeated reading of all transcripts followed by a detailed analysis of each text. The aim is to identify the nuclei of meaning concerning the research question to be labelled. Once the labelling work is completed, a protocol is constructed to record all the labels produced, to be able to compare the labels obtained with the meaning units to arrive at a definition of the labels that are as faithful as possible to the quality of the meaning units. Then the labels are grouped into categories, and similar categories are grouped into macro-categories, from which the text is structured.

Before starting the analysis, the interviews were audio-recorded and faithfully transcribed, word for word. Where possible, aspects of the non-verbal language, mimic expressions or gestures accompanying the speech were also reported.

Several strategies were implemented to ensure the rigor and reliability of the data, such as simultaneous data collection and analysis, and independent monitoring of the analysis by the researchers. In addition, the data analysis process was documented. Throughout the analysis, it was decided to keep track of any thoughts or judgements that arose to prevent judgement bias from distorting the analysis.

### Ethical considerations

All women freely chose to participate, and the timing of the interviews was agreed upon according to their preferences. Written informed consent to participate in the study, as well as the audio recording, was collected by the researcher upon first contact with the woman, and anonymity was guaranteed. Women were informed about the possibility of leaving the study at any time without compromising the quality of care.

Participants were also asked for some sociodemographic data for the definition of the sample. This information was recorded on an anonymous data collection form. Each participant was assigned a unique code, and all transcriptions were made without personal names. Names of maternity services and professionals were also anonymized.

## RESULTS

All the women were Italian and the average age was 33.5 years. The characteristics of the study participants are shown in Table 2. At the end of the maternity pathway: two women had received one-to-one care from their midwife only through pregnancy; five during pregnancy and after delivery; and three during pregnancy, labor and after delivery, one of which was assisted by the same midwife during delivery. Eight women received care from the public health service, and three from the private one.

**Table 2:** Participant characteristics (N=11)

*Characteristics*	*Categories*	*n*
**Maternal age** (years)	20–29	3
	30–39	6
	≥40	2
**Parity**	0	3
	1	4
	2	3
	3	1
**Education level**	PhD	1
	Master’s degree (2 years)	3
	Bachelor’s degree (3 years)	2
	High school diploma	5
**Working status**	Employed	9
	Unemployed	2

The analysis of the interviews led to the construction of a descriptive theory from which seven main topics emerged. Each macro-category comprises several categories and sub-categories (Table 3) as a coding system and explained hereunder. Next to each quote is the participant number, the timepoint of the interview, the week of pregnancy, and the pregnancy number.

**Table 3:** Coding system

*Topics*	*Categories*	*Sub-categories*
**Assessment of care**	Indicators of positive care	The progressive acquaintance allows one to establish a relationship of trust and help
		Personalized care
		Continuity of care considered reassuring
		Presence of a reference point
		Focus on positive elements and on childbirth physiology
		Organization of the caring path
		Assistance provided at home involving the family
		Professional network facilitating access to other caring paths
	Indicators of negative care	Improper and superficial care
		Lack of continuity of care
		Not enough time
		Midwife’s unavailability during weekends
		Assistance by telephone
**Communication assessment**	Indicators of positive communication	Continuous and immediate availability of staff
		Clear and accurate communication
	Indicators of negative communication	Absent or contradictory information
		Non-immediate availability of staff
		Inadequacy of e-mail communication
**How caring is perceived**	Indicators of good caring	Listening to and supporting women, providing holistic care
		Freedom to share thoughts, concerns and emotions
	Indicators of lack of caring	Lack of support, help and empathy
		Lack of consideration for the woman’s experience
**Emotional experiences of mother-to-be**	Positive emotional experiences	Feeling understood, accompanied and fully taken care of
		Feeling comfortable
	Negative emotional experiences	Concerns about child’s or one’s own health condition
		Concerns related to motherhood
**Thoughts of mother-to-be**	Self-awareness and beliefs	Right to the best possible care
		Need for assistance even in case of interruption of pregnancy
		Importance to consider the psychological and emotional dimensions as well as the physical one
		Maternal well-being is reflected in the child well-being
	Needs and desires	Being fully taken care of, on a physical and emotional level
		Maintaining continuity of care during and after childbirth
		Receive accurate and scientific information
		Non-medicalized motherhood
		Devote time to pregnancy
		Greater number of meetings with the midwife
**The decision-making process**	Possibility and impossibility of choice	Be allowed to choose the midwife who provides assistance
		Regret not to have the chosen midwife next to her during childbirth
		Sorry for not having the partner beside her
	Keeping options in view	The information received allows one to know the possible alternatives
		Feeling for an alternative, a possibility of choice
**The process of change**	Changes perceived by the mother	Acquiring information and new skills
		Greater self-confidence and perception of being more ready
		Greater peace of mind and self-consciousness
		Listening to and legitimizing one’s own needs

### First topic: Assessment of care

Women’s assessment of midwifery care enables an understanding of which elements maintain continuity of care and which interrupt it. Quality care, provided by trained and competent staff, guarantees the woman the necessary time and presence, which are central elements of continuity.

*Indicators of positive care*

Women positively assess the progressive relationship of acquaintance between them and the midwives, which allows a relationship of trust and help to be established. This relationship makes the woman feel cared for and accompanied:

*‘A relationship of trust has established, I never felt I wanted something else or something more than that. […] when I needed her, she was there.’* (Participant 7, second interview, 36-week gestation, second baby)

Knowing the woman, her story and her needs allows the midwife to personalize better the assistance she offers, becoming for the woman a point of reference to whom she can also turn to in the future, not only for maternity issues but concerning female well-being as a whole:

*‘If I need her also in the future, I will contact her, she has become a point of reference just like the general practitioner […] for me she is a point of reference.’* (Participant 1, third interview, 4 weeks after delivery, fourth baby)

Continuity of care is considered important since it provides a sense of assurance and peacefulness; the fact that midwifery cares to focus on positive elements and childbirth physiology is evaluated positively.

The good organization of the care pathway is also considered convenient and functional, as is the presence of a professional network that facilitates access to other care pathways. These elements make the woman feel taken care of, even beyond maternity-related aspects.

Finally, the accompaniment by a midwife offering assistance at home is reported as relevant. This not only allows the professional to get to know the maternal environment but also is considered an opportunity for women to experience maternity within the family in a shared way.

*Indicators of negative care*

The interviews revealed how the care that is considered inadequate and superficial could lead to a loss of trust and a questioning of the quality of the entire care process. Care is considered appropriate when more time is devoted to women. This, on the one hand, does not allow a relationship to be established with the professional and, on the other hand, does not allow the concerns that arise to be addressed.

The lack of continuity of care is something that is experienced negatively and is a cause of concern for the woman, who feels she is losing her point of reference.

This factor has been emphasized as a result of the COVID-19 health emergency:

*‘[After childbirth] It would have been nice to see her again, to say goodbye. This was not possible because of COVID-19. Yes, this is something I missed.’* (Participant 4, third interview, 4 weeks after delivery, third baby)

The women also identified the negative aspects of the midwife’s unavailability at weekends, which causes an interruption of the continuity of care. Similarly, receiving a phone call from the midwife instead of being personally visited, a change introduced because of the pandemic situation, was evaluated negatively:

*‘There was a moment when we met less frequently; we had planned meetings, but because of COVID-19 […] they didn’t take place, we still got in touch, by phone, by video calls but it’s not the same.’* (Participant 11, second interview, 36-week gestation, fourth baby)

### Second topic: Communication assessment

Communication-related aspects are another central element of continuity of care. Informing women, planning educational moments to respond to their needs and getting them involved in their care path are key to maintaining a therapeutic relationship. In case of need, the absence of information and midwifery availability leads to referral to the Emergency Department with an increase in improper accesses. During the study period, two women had unwarranted admission to the Emergency Department: one for an episode of sickness that occurred during the weekend when her midwife was unavailable, and the other for not having been adequately informed about a physiological change in the pregnancy.

*Indicators of positive communication*

Constant and immediate availability of midwifery staff is rated as essential, as it is considered a reassuring and helpful element:

*‘I got her phone number, we used to talk on the phone whenever I’m in need. This makes me feel safe, accompanied and supported … this is something I’d not do without. When I need, I call and find what I need.’* (Participant 10, first interview, 25-week gestation, third baby)

Indicators of positive communication include clear and accurate communication tailored to the listener. Adopting a mode of communication that considers individual needs makes the person feel understood, not judged, and involved, and the level of understanding is also higher.

*Indicators of negative communication*

In contrast, the absence of information, as well as discordant and contradictory information, is evaluated negatively and makes the woman feel not only that she is not taken care of but also that she is not sufficiently prepared to face the subsequent stages of motherhood.

Likewise, the lack of immediate availability of professionals contributes to a negative assessment of the entire path, making mothers feel lost and abandoned:

*‘I had called her several times and could not find her, so I sent her an e-mail, but I happened to write to her and get a reply after a week. […] A mother feels a bit abandoned.’* (Participant 8, second interview, 36-week gestation, first baby)

In this regard, communication via e-mail is considered inadequate and does not facilitate the free expression of thoughts, questions, and state of mind.

### Third topic: How caring is perceived

For women, the perception of being cared for, listened to, and supported in their needs throughout the maternity journey is paramount.

*Indicators of good caring*

The woman evaluates being listened to by and support from the midwife as indicators of good caring. The midwife cares for the woman physically, emotionally, and relationally, considering her experience, makes the woman feel reassured and taken care of.

Accompaniment and emotional support enable one to accept and overcome anxieties and fears. Furthermore, having the opportunity to freely confront one’s midwife and to share thoughts, emotions, doubts and worries without feeling judged, allows the woman’s innate skills to emerge:

*‘A dialogue is going on, she is not just somebody telling you how it has to be done, but there’s a dialogue, it’s about what you think, what you feel, and so she brings out my competence.’* (Participant 5, third interview, 4 weeks after delivery, second baby)

*Indicators of lack of caring*

Indicators of lack of caring include women’s perception of a lack of support and tools to help them cope with difficulties. Perceiving this lack of caring and empathy from the staff, makes women feel unseen and disrespected. Women remember such incidents even long afterwards as unpleasant and impactful for them, as well as when they perceive that their previous experiences did not get any consideration:

*‘What annoyed me was the way she said it to me, without giving any explanations, and when I told her “what if I take responsibility for this” she laughed in my face and said “if I tell you to do it, you will do it”.’* (Participant 1, first interview, 25-week gestation, fourth baby)

### Fourth topic: Emotional experiences of the mother-to-be

Investigating the emotional experiences that emerge during maternity provides a more accurate comprehension of how to support and accompany women by providing them with the necessary tools and information so that negative experiences move aside for positive ones.

*Positive emotional experiences*

Feeling understood, accompanied, and taken care of on a global level is what makes the woman feel at the center of care, safe and quiet:

*‘It’s like maternity, I mean that you’re experiencing pregnancy and meanwhile the midwife is like a supportive mother. This means to be cared for.’* (Participant 7, second interview, 36-week gestation, second baby)

The woman in the relationship with the midwife can feel at ease, feels that even her pain is legitimate, as are her fears and worries. This condition is experienced as a resource.

*Negative emotional experiences*

During pregnancy, one of the main maternal concerns that emerged was related to the baby’s health or her own. Due to the health emergency, even the hospital setting caused concerns to the extent that it was no longer perceived as a safe place:

*‘I had doubts about whether to do the morphology scan or not, mainly because of COVID, I was afraid it wasn’t worth moving from home.’* (Participant 3, first interview, 26-week gestation, second baby)

Other fears are related to maternity itself, either in relation to the pregnancy, the time of delivery, and after childbirth; again, the health emergency increased the women’s concern of being left alone:

*‘The only concern I have right now is giving birth alone because of this COVID-19.’* (Participant 8, first interview, 24-week gestation, first baby)

### Fifth topic: Thoughts of the mother-to-be

Knowing what women think and need the most helps to structure care paths that not only take care of these needs but also make it possible to personalize care.

*Self-awareness and beliefs*

A certain degree of maternal awareness emerged from the interviews, such as the right to receive the best possible care and assistance, to be guaranteed even in spontaneous abortion. Women also express certain beliefs, such as the importance of considering not only the physical factors but also the psychological and emotional dimensions:

*‘Psychological wellbeing is just as important as physical wellbeing, getting attention to the former is harder. It is more difficult to find someone who will listen to you, but what I feel and think has the same value as what happens on a physical level.’* (Participant 1, first interview, 25-week gestation, fourth baby)

Besides, women believe that maternal well-being is reflected in the child’s well-being, both during pregnancy and after birth: for this reason, being taken care of by a midwife is considered a valuable experience that may have positive effects on the mother and, indirectly, on the baby.

*Needs and desires*

The women expressed various needs, such as the need for global care by the midwife, not only from a physical point of view but also on an emotional level. Strong is the desire to maintain continuity of care even during delivery, moments of hospitalization and after childbirth, precisely because of the knowledge and relationship of trust established:

*‘Having the same midwife accompanying you in pregnancy and during childbirth would be a value.’* (Participant 5, first interview, 24-week gestation, second baby)

Other needs that emerged, previously discussed, are to receive scientific and accurate information and to be able to devote time to taking care of oneself and of the pregnancy, which women want to experience in a non-medicalized manner. Midwife visits are experienced as moments of well-being, and for this reason, some women expressed the desire to meet their midwife even more often.

### Sixth topic: The decision-making process

Continuity of care aims at ensuring women’s involvement in their care path, promoting their awareness and freedom of choice through support, education, and information.

*Possibility and impossibility of choice*

In the context of the decision-making process, some possible and some not possible choices emerged. Among the possible decisions, being able to choose the midwife who will accompany her through the maternity journey is seen by the woman as a relevant element, in terms of relationship, compared to being cared for by someone not chosen. Nevertheless, such a choice is often not possible, and it may not be possible for the woman to have the midwife who followed the pregnancy beside her even during childbirth. This, which women experience as an inconvenience, has been accentuated by the health emergency:

*‘I’d have really loved it if she [the midwife who took care of me during pregnancy] had come to the hospital, and I am sorry that she couldn’t come because of this COVID situation.’* (Participant 3, second interview, 36-week gestation, second baby)

The pandemic situation also prevented the involvement of women’s partners during pregnancy and hospital visits, causing women worries, regret and a sense of loneliness.

*Keeping options in view*

Fundamental to the decision-making process was the information provided by the midwife, who helps women to know the possible alternatives and accompanies them in making choices. Thanks to this accompaniment, women feel they have an alternative. Taking responsibility for their own choices becomes a significant factor in taking care of themselves and their baby:

*‘For me, knowing I had an alternative, a possibility of choice, by taking my responsibility, was important.’* (Participant 6, first interview, 24-week gestation, first baby)

### Seventh topic: the process of change

Quality care enables women to acquire new information and skills by providing the necessary tools to cope with the changes brought by maternity.

*Changes perceived by the mother*

The women emphasized the value of acquiring new information and additional skills in managing their pregnancy, and recognizing signs of their baby’s health during and after childbirth. They reported increased confidence in themselves and their abilities, feeling more ready to face the following maternity stages and experiencing events with more significant serenity and awareness:

*‘Having the possibility to deal with issues and solve doubts during pregnancy has enabled me to arrive more ready for what happened thereafter, with more knowledge and more self-confidence.’* (Participant 2, third interview, 4 weeks after delivery, second baby)

Besides, having learnt to listen more to themselves, justifying their needs has led them to accept even the most difficult maternity moments, making it easier to ask for help when needed, during and after pregnancy.

*Evolution of the woman-midwife relationship along the maternity journey*

As three interviews were conducted with each woman at different times during maternity, the evolution of the care relationship could also be analyzed. The interviews revealed how certain elements such as listening, knowledge, participation, and availability, can help establish a caring relationship:

*‘I feel more reassured now, compared to the first times, a relationship has been gradually built up. […] She understood that I needed reassurance and accepted my need … there was no confidence at the beginning, which did not allow me to trust her. That relationship of knowledge was missing.’* (Participant 4, second interview, 36-week gestation, third baby)

In contrast, elements such as superficiality in the assistance, lack of response or disinterest can deeply damage this relationship:

*‘I visited with another midwife because mine was ill, and on that occasion, I discovered that I had had a urinary tract infection and had not been treated. I assumed that I could trust her, but it wasn’t like that … you wonder if everything else was done correctly.’* (Participant 8, second interview, 36-week gestation, first baby)

## DISCUSSION

The aim of this research was to analyze in depth the concept of continuity of care from pregnancy to after childbirth. The results show how continuity of care is a multidimensional concept integrating aspects that have to do with assistance, caring, and experiences, thoughts, choices, and changes.

Each of these elements plays a role within a continuity of care pathway, and the lack of one may undermine the entire process.

Previous studies, though carried out in different contexts, highlighted the complexity of this phenomenon and the importance of continuity of care for women during pregnancy^16,17^.

One of the main findings of this research, which should be further investigated, is the extent to which quality care enables mothers-to-be to acquire new information and skills, feel more aware and increase their confidence in themselves and their abilities. Although other studies^18,19^ have found that continuity of care may develop women’s empowerment, this research reveals that the newly acquired skills are a benefit during pregnancy and in the postnatal period, and in the relationship with the baby. Listening to oneself, justifying one’s needs and being capable of asking for help, are key elements in protecting maternal and, consequently, neonatal health, especially in a critical period such as the post-partum period. These results are very relevant even from the perspective of preventing postnatal depression.

Interviews also show that care must be provided by competent and adequately trained professionals capable of establishing a relationship of trust. Motivated, experienced personnel with great expertise and communication skills play a significant role in maintaining continuity of care^17^. Trust emerges as a key element of the relationship in several studies^17-19^. Other research^17,19^ shows how poor care undermines women’s trust in the professional by compromising the continuity relationship.

To maintain continuity of care, a strong relationship must be established between the woman and the midwife, a relationship that should continue through all stages of motherhood, from pregnancy to postpartum^20^. The relationship is a central element, also highlighted in previous studies^16,21^. Such a relationship also established through a progressive acquaintance, allows the midwife to take charge of the woman physically and mainly emotionally. Dahlberg and Aune^19^ notice how being taken care of on a holistic level is a need for the woman that allows the professional to understand the deepest maternal needs and aims at developing a supportive and helpful relationship, and personalized care as much as possible based on the mother’s needs and values^19^. Some authors^17,19^ underline how disregarding the person and her values may disrupt the continuity of care and negatively affect the maternity experience. In contrast, respectful professionals who avoid inappropriate behavior and respect women’s choices succeed in maintaining a positive continuity of care relationship^17^.

Midwifery care is aimed at preserving the physiology of pregnancy, avoiding medicalization and unnecessary or inappropriate interventions. This is consistent with findings from other studies, which highlighted that if care is based on the physiology of birth and succeeds in responding to women’s different needs, it may also improve related outcomes^17,21^. Overtreatments, unnecessary procedures and early hospitalizations may instead disrupt the relationship of trust and continuity^17^, leading to unfavorable outcomes in the maternity experience.

The interviews revealed women’s need to be at the center of the care relationship with their needs, experiences, thoughts and expectations. Women are not the object of care but active and aware subjects demanding to take part in the care process and to be able to choose what is best for themselves and their babies. Participating in the decision-making process and being enabled to take decisions are considered fundamental rights of women^17,22,23^ and should be supported. In this regard, a key role is played by communication, which must be clear, accurate and tailored to the target audience. The importance of constant information exchange has also been reported by previous studies and implies good communication skills and strategies on the practitioner’s part^16,17,24^. Bagheri et al.^17^ additionally highlight the importance of information and lifelong education, which may enhance self-care capability, self-confidence, and self-esteem^17^. In contrast, professionals’ lack of communicative skills and inability to provide adequate information may cause the disruption of continuity and increase unwarranted admission to healthcare facilities^17^. This aspect emerged in this research too.

Having access to scientific, adequate, and accurate information on the one hand and getting a chance to discuss it freely without prejudice, on the other, enable women to make informed choices and feel more responsible, increasing their competence. Support, encouragement and absence of judgement are highly valued by women^19,21^.

Another key aspect underlined in this study is the possibility of reaching one’s midwife at any time, including weekends and public holidays. Rapid access is essential to maintain continuity of care, as found in previous studies^16,17^, and avoid unwarranted admission to hospitals.

In this regard, adequate means for women to contact their midwives must be ensured, so more than an e-mail address is needed and a direct telephone contact to call in case of need is essential. Fereday et al.^21^ highlight that women feel reassured when they know they can call their midwives at any time, an aspect that also emerged clearly in this research.

The midwife should also be available during hospitalization, childbirth and postpartum. This is consistent with Fereday et al.^21^ who underlined that such continuity responds to a women’s expectations. They feel a strong need to have a person they can trust and rely on, who knows their history, experiences and needs. Such connection is strengthened by the care provided at the woman’s home, as also shown in the study by Bagheri et al.^17^. Here, the midwife can get to know the environment where the mother lives and the entire family can get involved. Moreover, Bagheri et al.^17^ indicate a mother’s home as a key element to recognize situations of economic or social difficulty.

### Strengths and limitations

Although the results of this study are firmly anchored in the context and area in which the study was conducted and the organizational models in place, this research analyzed a considerable number of interviews, which can be considered a strength. Besides, since the research was carried out during a health emergency, which also involved women and their access to maternity-related services, some of the repercussions of the COVID-19 pandemic on women and their experience of continuity of care emerged. Giving preference to phone calls rather than personal visits and preventing women’s partners from accessing hospital facilities, and leaving women without the possibility of having a person of their choice at their side, generated anxiety and fear. This issue was also highlighted in the editorial by Coxon et al.^25^, where it was underlined that the organizational changes introduced during the pandemic raised concerns about women’s right to get supported by partners during and after childbirth. In several cases, continuity of care was also interrupted for this reason.

Despite the limitation of a small number of participants, important issues emerged that could be taken into consideration both with a view to the implementation of the midwifery continuity of care model and the reorganization of services in health emergencies.

## CONCLUSIONS

The concept of continuity of care is rather complex since it integrates several elements.

One of the main findings of this research is the role of new skills acquired by the woman assisted in midwifery continuity of care. Taking care of oneself and knowing how to ask for help are fundamental elements in maintaining a good level of health and living the maternity experience positively. Professionals play a central role in establishing a beneficial and empowering relationship.

From the perspective of prevention and protection of maternal and child health, both in the short- and long-term, it becomes essential to focus on the development of maternal competencies also through adequate training of professionals in relational and empathic skills as well as clinical ones.

This may be possible by implementing midwifery-led continuity of care pathways according to evidence of the effectiveness of the midwifery-led continuity of care model and international and national recommendations. These paths should take women’s experiences into account, ensuring an appropriate and flexible organizational system capable of responding to women’s needs throughout the entire maternity journey. Attention should also be given to creating the conditions for good clinical practice to be maintained even during health emergencies.

## Data Availability

The data supporting this research are available from the authors on reasonable request.
